# Health-related quality of life during early aggressive treatment in patients with polyarticular juvenile idiopathic arthritis: results from randomized controlled trial

**DOI:** 10.1186/s12969-019-0370-1

**Published:** 2019-12-16

**Authors:** Maarit Tarkiainen, Pirjo Tynjälä, Paula Vähäsalo, Liisa Kröger, Kristiina Aalto, Pekka Lahdenne

**Affiliations:** 1 Children’s Hospital, Helsinki University Central Hospital, Helsinki, Finland; 2 Pediatric Research Center, University of Helsinki, Helsinki, Finland; 3 Poison Information Centre, Helsinki University Central Hospital, Helsinki, Finland; 4 Department of Children and Adolescents, Oulu University Hospital, Oulu, Finland; 5 PEDEGO Research Unit, University of Oulu, Oulu, Finland; 6 Medical Research Center Oulu, Oulu University Hospital and University of Oulu, Oulu, Finland; 7 Department of Pediatrics, Kuopio University Hospital, Kuopio, Finland; 8 HUS, P.O. Box 705, 00029 Helsinki, Finland

**Keywords:** Juvenile Idiopathic Arthritis, Health-related Quality of Life, Early aggressive treatment, DMARDs, Infliximab

## Abstract

**Background:**

Juvenile Idiopathic Arthritis (JIA) may cause significant impairment in health-related quality of life (HrQoL), despite effective therapies. The aim of this study was to assess HrQoL during first-year treatment in patients with new-onset polyarticular JIA, and to compare treatment strategies.

**Methods:**

In ACUTE-JIA Study, 60 patients with new-onset JIA were randomized to receive either infliximab with methotrexate (IFX+MTX); a triple therapy of methotrexate, hydroxychloroquine, and sulfasalazine (Triple); or methotrexate monotherapy (MTX). Efficacy was measured with American College of Rheumatology pediatric (ACRp) score, and juvenile arthritis disease activity score (JADAS). HrQoL was evaluated with Child Health Questionnaire (CHQ), which includes physical and psychosocial summary scores (PhS and PsS). Linear mixed models were utilized to compare groups over time.

**Results:**

In the whole group of 60 patients, mean physical summary score (PhS) improved from 26.2 (SD 8.7) at week 0 to 49.7 (SD 13.2) at week 54 (p=0.046). Mean improvement of PhS was 20.3 (95% CI -15.5 to 56.2); 22.6 (-19.5 to 64.7); and 26.6 (-12.1 to 65.3) in IFX+MTX, Triple, and MTX, respectively. Changes in psychosocial summary score (PsS) were smaller: from 51.0 (SD 8.5) to 54.7 (6.3) (*p*=0.019) in all patients. No differences between the three treatment groups were detected in either of the measures. In multivariate analyses, Child Health Assessment Questionnaire (CHAQ), pain VAS, and time spent in inactive disease contributed to improvement in PhS; gender and CHAQ to PsS.

**Conclusions:**

HrQol improved during the first year on therapy for JIA irrespective of the treatment strategy. The timing of change in the different dimensions of HrQoL varied; improvement occurred earlier in physical than psychosocial domains of HrQol.

**Trial registration:**

This study was registered within the Hospital District of Helsinki and Uusimaa (http://www.hus.fi) clinical trials, number 211864 in October 2002, and later on with ClinicalTrials.gov, number NCT01015547.

## Background

Juvenile idiopathic arthritis (JIA) is a chronic condition, which may cause disability and functional impairment. JIA is treated with non-steroidal anti-inflammatory drugs, synthetic disease-modifying anti-rheumatic drugs (DMARDs), glucocorticoid injections, and in refractory disease, with biologic drugs.

Health-related Quality of Life (HrQoL) is a multi-dimensional health outcome concept, which takes into account perspectives of well-being, physical health, and psychological state, as well as the surrounding culture and environment [1]. In health technology assessments of new treatment approaches, HrQoL is an important component in the measurement of efficacy. In JIA, baseline HrQoL seems to have an independent role in predicting disease activity and functional disability [2].

Patients with JIA often have impaired HrQoL [3]. On the other hand, effective therapies targeting disease activity have improved HrQoL [4]. Besides disease activity, other factors such as pain, disability, and burden of medication can affect HrQoL [5].

In patients with new-onset JIA, only a few studies have evaluated the effects of treatment on HrQoL. In a recent german inception cohort, psychosocial health reached the level of normal population at 18 months from onset, whereas a significant difference in physical health remained [4]. Disease activity, functional disability [4, 6], parent or patient global assessment of wellbeing, and pain [4] seem to play a major role in HrQoL. Yet it is not known how safety and efficacy of different medications affect HrQoL.

In this study, we aimed to assess HrQoL during the first-year of early aggressive treatment in patients with polyarticular JIA, and the impact of different treatments on HrQoL.

## Methods

This study was part of the ACUTE-JIA Study, in which sixty patients with new-onset polyarticular JIA were randomized into three treatment arms: infliximab with methotrexate (IFX+MTX); a combination of methotrexate, hydroxychloroquine, and sulphasalazine (Triple), or methotrexate monotherapy (MTX). Study protocol has been described in detail previously [7]. In brief, patients entered the trial very early in the disease course, less than 6 months from disease onset. During the first year, patients underwent altogether eight study visits. During the first year, patients underwent altogether eight study visits. At these visits, disease activity was measured with American College of Rheumatology pediatric (ACRp) scores, and juvenile arthritis activity score (JADAS). Medication was adjusted according to the study protocol, aiming to a minimum of ACRp 30 improvement. At seven visits (weeks 0, 6, 12, 24, 36, 48, and 54) HrQoL was measured with parents’ proxy reports on Child Health Questionnaire (CHQ)-PF50. Pain was measured on a visual analog scale (0-100 mm). The preliminary Wallace criteria [8] were used to define clinically inactive disease (CID). Intra articular corticosteroids (GCs) were allowed as symptomatic treatment, whereas systemic GCs were not allowed.

CHQ is an international, validated, and generic HrQoL survey. CHQ-PF50 has 50 questions that together produce 15 health concepts (Global Health, Physical Functioning, Role/Social Limitations - Emotional/Behavioral or Physical, Bodily Pain/Discomfort, Behavior, Global Behavior Item, Mental Health, Self Esteem, General Health Perceptions, Change in Health, Parental Impact -Emotional or Time, Family Activities and Cohesion), each producing values between 0-100. It also constitutes two summary scores Physical Summary Score (PhS), and Psychosocial Summary Score (PsS), which have been standardized with a linear T-score transformation (mean 50; one standard deviation of age and sex matched healthy children is 10, changes of 5 or 8 are considered moderate or large). In a normal U.S. population sample, mean PhS is 53.0 (SD 8.8), and PsS 51.2 (SD 9.1) Higher scores indicate for better health [9].

The CHQ values were calculated as advised by the publisher [9]. For missing items, scores were imputed if at least half of the items in the scale were completed, as advised. For patients who discontinued medication, the last observation on intended treatment was carried forward.

### Statistical analyses

Differences between groups at baseline were tested with one-way analysis of variance, Kruskal-Wallis test, or Chi square, as appropriate. Paired samples t-test was used to test differences in summary scores between time points. A linear mixed model was developed to investigate changes in summary scores over time, and contributing factors. First, demographic and variables related to treatment and disease activity were selected to perform separate univariate analyses. Second, significant variables were used in the final linear mixed model.

IBM SPSS Statistics Version 24 (IBM Corp., Armonk, NY, USA) was used for all statistical analyses.

## Results

Parents of all 60 patients filled the CHQ in the beginning of the study. Of these, 20 patients remained on intended treatment on IFX+MTX, 16 on Triple, and 11 on MTX. Patients in IFX+MTX achieved and remained in inactive disease more often than in other groups. Differences between Triple and MTX were not statistically significant (7). Patients in MTX had more infections and transient elevations of liver enzymes than in other groups. Nausea was more common in Triple. 3 patients in Triple and 8 in MTX discontinued due to inefficacy, one patient in Triple and one in MTX due to an adverse event. At baseline, compared with other treatment groups, patients on IFX+MTX had lower CHAQ, JADAS, and pain. PhS and PsS levels were similar in all treatment groups. (Table 1)

**Table 1:** Baseline characteristics of 60 patients with new-onset polyarticular JIA

	IFX+ MTX	Triple	MTX	p
Age (yrs)	10.5 (4.5—14.9)	8.2 (4.8—13.3)	10.1 (4.0—14.4)	0.06
Female (%)	14 (70)	14 (70)	11 (55)	0.21
Time (months) from disease onset	0.82 (0.0-2.7)	1.77 (0.03—7.0)	1.18 (0.03—7.1)	0.20
Rheumatoid factor positive	0	1 (5)	0	
JIA subtype				
Polyarthritis, seronegative	19 (95)	16 (80)	15 (75)	
Enthesitis-related arthritis (%)	1 (5)	3 (15)	4 (20)	
Polyarthritis, seropositive	0	1 (5)	0	
Psoriatic arthritis	0	0	1 (5)	
CHAQ	0.49 (0—1.75)	0.71 (0—2.00)	1.07 (0—1.88)	0.01
JADAS10	17.1 (6.9—23.4)	20.1 (9.8—29.8)	21.9 (11.4—30.6)	0.01
Pain-VAS	24.5 (0—90)	37.6 (4—78)	46.7 (2—85)	0.02
PhS	28.2 (21.4—35.1)	28.8 (17.9—39.6)	21.9 (12.8—30.9)	0.46
PsS	51.4 (27.1—58.5)	48.9 (37.1—62.8)	52.8 (40.2—65.8)	0.28

Values are presented as mean (range), unless otherwise stated. IFX+MTX: infliximab+methrotrexate; Triple: Triple therapy of sulphasalazine, hydroxychloroquine, and methotrexate; MTX: methotrexate; *CHAQ* child health assessment questionnaire, *JADAS* juvenile arthritis disease activity score, *VAS* visual analoque scale, *PhS* Physical summary Score, *PsS* Psychosocial summary Score

**Fig. 1:**
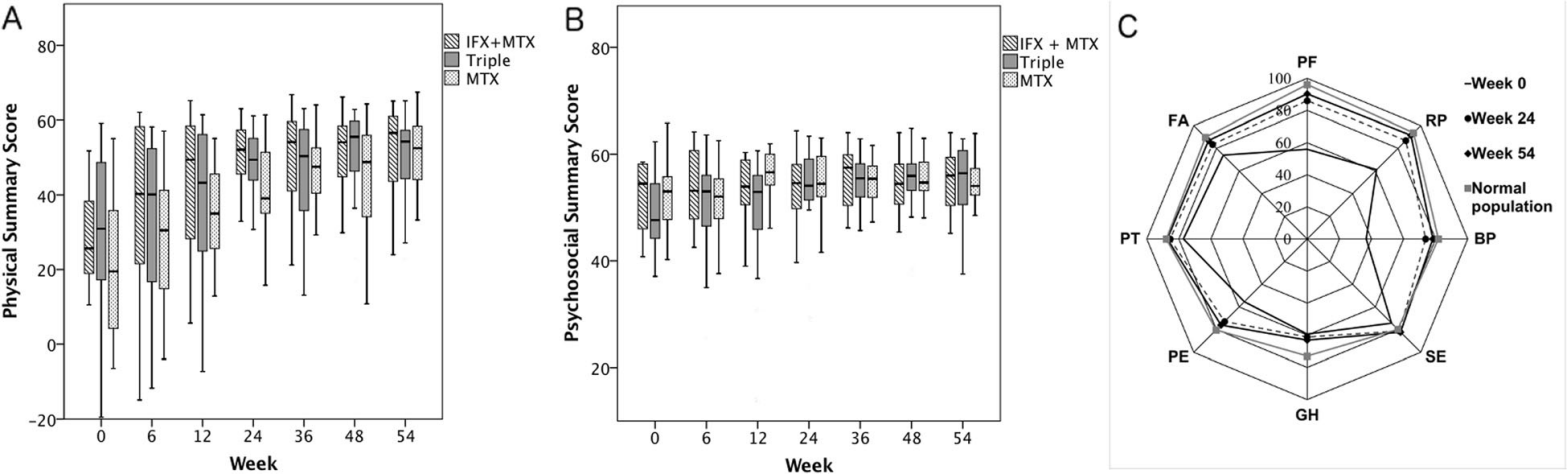
Evolution of health-related quality of life during first year in patients with JIA

Mean (SD) PhS at week 54 was 49.9 (15.5) on IFX+MTX; 49.9 (12.0) on Triple; and 49.3 (12.9) on MTX, (p=0.98). Changes in PhS in the total cohort were significant between weeks 0 to 6 (p= 0.023); 6 to 12 (p=0.020); and 12 and 24 (p=0.004) (Figure 1). Mean PsS at week 54 was 54.9 (6.0) on IFX+MTX; 55.0 (6.8) on Triple; and 54.1 (6.3) on MTX, (*p*=0.89). There were no significant changes between consecutive weeks, but scores at weeks 24, 36, 48, and 54 were significantly higher than at week 0. (Fig. 1a and b)

In the mixed model, Child Health Assessment Questionnaire (CHAQ), pain, and cumulative time spent in CID prior to each study visit contributed significantly to PhS values (Table 2). For PsS, gender and CHAQ remained significant contributors in the mixed model (Table 3).

**Table 2:** Factors contributing to Physical Summary Score (PhS) in 60 patients with new-onset polyarticular juvenile idiopathic arthritis

	Univariate		Mixed Model	
	β (95% CI)/*Mean PhS (95% CI)*	*p*	β (95% CI)	*p*
Gender (mean)		0.14		
Female	*44.5 (40.7 to 48.3)*			
Male	*39.9 (34.9 to 44.8)*			
Time of visit (weeks)^a^		<0.001		<0.001
0	*26.22 (22.04 to 30.39)*		*35.46 (30.91 to 40.04)*	
6	*33.53 (29.43 to 37.63)*		*38.38 (34.30 to 42.46)*	
12	*38.69 (34.59 to 42.79)*		*39.95 (36.34 to 43.55)*	
24	*47.98 (43.76 to 52.19)*		*43.57 (41.63 to 45.86)*	
36	*48.07 (43.81 to 52.32)*		*44.62 (42.04 to 47.19)*	
48	*49.80 (45.54 to 54.05)*		*44.29 (41.07 to 47.51)*	
54	*51.66 (47.40 to 55.91)*		*46.51 (43.66 to 49.35)*	
Age at onset	-0.01 (-0.97 to 0.95)	0.98		
Treatment (mean) ^b^		0.043		0.419
IFX+ MTX	*46.2 (41.0 to 50.5)*	0.046^c^	*40.19 (36.98 to 43.42)*	
Triple	*45.5 (40.4 to 50.5)*	0.065^d^	*42.70 (39.85 to 45.55)*	
MTX	*38.7 (33.5 to 43.9)*		*42.65 (39.53 to 45.78)*	
JADAS10	-1.258 (-1.45 to -1.06)	<0.001	-0.347(-0.72 to 0.024)	0.067
Pain-VAS	-0.432 (-0.49 to -0.38)	<0.001	-0.204 (-0.28 to -0.12)	<0.001
Corticosteroid use^e^	-0.473 (-0.64 to -0.30)	<0.001	-0.004 (-0.019 to 0.10)	0.554
Time in remission^f^	0.204 (0.073 to 0.334)	0.003	0.172 (0.05 to 0.29)	0.005
CHAQ	-23.25 (-26.1 to -20.4)	<0.001	-11.2 (-15.8 to -6.67)	<0.001
CHAQ*JADAS			0.087 (-0.29 to 0.47)	0.658

For numerical factors, Coefficient beta indicated; for categorical values mean PhS value indicated.

Sidak correction for multiple comparisons used. Factors with *p*-value <0.05 in univariate analyses were selected for multivariate mixed model. Values shown in Mixed Model column have been adjusted with other variables in the model. IFX+MTX: infliximab plus methotrexate; Triple: methotrexate, hydroxychloroquine, and sulfasalazine; *MTX* methotrexate monotherapy, *JADAS* juvenile arthritis disease activity score, *VAS* visual analoque scale, *CHAQ* child health assessment questionnaire, *PhS* Physical summary Score. ^a^Values present mean (95% CI) PhS in all patients at each time point ^b^Values present mean (95%CI) PhS at all time points in each treatment group ^c^IFX+MTX vs MTX; ^d^Triple vs MTX; for IFX+MTX vs Triple *p*=0.84; ^e^Amount of corticosteroids per body weight (intra-articular or oral) before individual study visit, adjusted for 6 weeks’ time (mg/kg); ^f^Cumulative time in remission since onset, *interaction term

**Table 3:** Factors contributing to Psychosocial Summary Score (PsS) in 60 patients with new-onset polyarticular juvenile idiopathic arthritis

	Univariate		Mixed Model	
	β (95% CI)/*Mean PsS (95% CI)*	*p*	β (95% CI)	*p*
Gender (mean)		<0.001		
Female	*54.66 (53.78 to 55.54)*			
Male	*51.12 (49.98 to 53.27)*			
Time of visit (weeks)		0.036		
0	*51.00 (49.10 to 52.90)*			
6	*52.02 (50.15 to 53.89)*			
12	*52.76 (50.89 to 54.63)*			
24	*53.81 (51.94 to 55.68)*			
36	*54.48 (52.58 to 56.38)*			
48	*54.70 (52.82 to 56.58)*			
54	*54.66 (52.77 to 56.54)*			
Age at onset	0.134 (-0.92 to 0.36)	0.245		
Treatment (mean)		0.113		
IFX+ MTX	*53.72 (52.4 to 55.0)*	*0.971a*		
Triple	*52.4 (51.1 to 53.5)*	*0.139b*		
MTX	*54.1 (52.8 to 55.3)*			
JADAS10	-0.251 (-0.378 to -0.125)	<0.001	-0.038 (-0.24 to 0.16)	0.701
Pain-VAS	-0.084 (-0.118 to -0.051)	<0.001	0.001 (-0.055 to 0.58)	0.96
Corticosteroid used	-0.005 (-0.014 to 0.003)	0.193		
Time in remissione	0.009 (-0.035 to 0.054)	0.679		
CHAQ	-5.710 (-7.117 to -4.303)	<0.001	-5.07 (-7.85 to -2.29)	<0.001
CHAQ*JADAS			-0.116 (-0.309 to 0.077)	0.237
CHAQ*Pain-VAS			0.047 (-0.011 to 0.105)	0.115

For numerical factors, Coefficient beta indicated; for categorical values mean PsS value indicated.

Sidak correction for multiple comparisons used. Factors with *p*-value <0.05 in univariate analyses were selected for multivariate mixed model. Values shown in Mixed Model column have been adjusted with other variables in the model. IFX+MTX: infliximab plus methotrexate; Triple: methotrexate, hydroxychloroquine, and sulfasalazine; *MTX* methotrexate monotherapy, *JADAS* juvenile arthritis disease activity score, *VAS* visual analoque scale, *CHAQ* child health assessment questionnaire, *PsS* Psychosocial summary Score. ^a^IFX+MTX vs MTX; ^b^Triple vs MTX; for IFX+MTX vs Triple *p*=0.318; ^d^Amount of corticosteroids per body weight (intra-articular or oral) before individual study visit, adjusted for 6 weeks’ time (mg/kg); ^e^Cumulative time in remission since onset, *interaction term

Of the single domains of HrQoL; bodily pain (BP), role or social limitations due to physical reasons (RP), physical functioning (PF), global health perception (GH), and parental emotional impact (PE) differed from normal population at week 0. Other domains did not differ from normal population. At week 54, patients reached normal population in other domains, except GH (Fig. 1c)

## Discussion

In the present intention-to-treat analysis of new-onset polyarticular JIA, HrQoL improved irrespective of the treatment strategy, although the timing of change in the dimensions of HrQoL varied. This suggests that early aggressive treatment of JIA either with biologics or synthetic DMARDs makes it possible to control imminent impairment of HrQoL.

Physical condition improved soon after the onset of aggressive therapy, simultaneously with the decrease in disease activity. In a recent study from Canada, improvement in HrQoL occurred later than in other disease outcome measures, several years after improvement of disease activity [5]. However, in that study only 10% of patients received biologics. In the current study, each of CHAQ, pain VAS, and time spent in CID were associated with improvement in physical condition. This underlines the significance of controlling disease activity to ensure good quality of life.

The changes of psychosocial condition in this study were minor, and significant improvement over time compared to the situation at onset of therapy could not be seen until week 24. Usually, psycho-social components of QoL have been affected to lesser extent than physical components [3, 4]. In line with the present study, previous studies utilizing CHQ or SF-36 have found only small changes in PsS [10]. In a recent study on abatacept, measured with CHQ, patients with JIA reported better PsS values than healthy normal population, already in the beginning of treatment [11].

In this study, the subdomain parental emotional impact remained below normal population at the end of the first year. Measurements in this study were parent proxy-reports, which however have shown similarities [12] with the juvenile patients’ self-reporting. In a previous study on patients with new-onset polyarticular JIA, parental emotional scores have been a significant predictor of proxy-reported HrQoL [4]. These findings indicate that it is important to pay attention to parents’ emotions to enable holistic approach for patient care in JIA.

In this study, pain contributed to physical aspects of QoL. This was also demonstrated previously [3, 4, 13]. This suggests that instead of HrQoL questionnaires with multiple items, pain VAS could possibly be used in clinical work as a rapid tool to assess patient’s well-being.

In long-term follow-up studies, patients with JIA have shown poorer HrQoL than their peers, even despite their low disease activity [6, 10]. One reason for this might be the less effective treatment available at the time of disease onset of these studies. The current study demonstrated that despite the differences in the safety and efficacy profiles of biologics and DMARDs [14, 15], these treatments are equal in improving HrQoL, when used in a treat-to-target manner.

A limitation of this study was the small sample size, which decreased the power to detect differences between treatment groups. Despite the fact that there were differences in the efficacy (IFX +MTX was more effective than other treatments), no differences were detected in the evolution of HrQoL. This might be due to the small sample size, and the differences at baseline. HrQoL was measured during the first year from disease onset. Longer follow-up time may be needed to reveal the effects of early aggressive therapy and other contributing factors on HrQoL. The effects of non-medical conditions, such as social support and school absence, were not measured.

To the best of our knowledge, this is the first study comparing HrQoL between biologic and synthetic DMARD therapy. In the future, a long-term follow-up study on HrQoL, assessing the effects of early aggressive treatment and factors associated with HrQoL might direct treatment decisions towards better patient-reported outcomes.

## Conclusions

In conclusion, quality of life improved in all treatment groups during the first year from onset of polyarticular JIA. Despite their different efficacy and safety profiles, infliximab and synthetic DMARDs, when used early and aggressively, were effective in improving health-related quality of life.

## Data Availability

The datasets used and/or analysed during the current study are available from the corresponding author on reasonable request.

## Abbreviations

ACRp: American College of Rheumatology pediatric (score)
BP: Bodily pain
CHAQ: Child health assessment questionnaire
CHQ: Child health questionnaire
CID: Clinically inactive disease
DMARDs: Disease-modifying anti-rheumatic drugs
GH: General health perceptions
HrQoL: Health-related quality of life
JADAS: Juvenile arthritis disease activity score
JIA: Juvenile Idiopathic Arthritis
PE: Parental emotional impact
PF: Physical functioning
PF-50: Patient form 50
PhS: Physical summary score
PsS: Psychosocial summary score
RP: Role or social limitations due to physical reasons
SF-36: Short form health survey
VAS: Visual analogue scale
